# Regulation of mRNA and miRNA in the response to *Salmonella enterica* serovar Enteritidis infection in chicken cecum

**DOI:** 10.1186/s12917-022-03522-y

**Published:** 2022-12-14

**Authors:** Xiuxiu Miao, Lewen Liu, Liying Liu, Geng Hu, Guixian Wu, Yuanmei Wang, Yanan Zhao, Jingchao Yang, Xianyao Li

**Affiliations:** 1 College of Animal Science and Technology, Shandong Provincial Key Laboratory of Animal Biotechnology and Disease Control and Prevention, Shandong Agricultural University, Tai’an, 271018 China; 2 College of Life Sciences, Shandong Agricultural University, Tai’an, 271018 China; 3 Shandong Animal Husbandry General Station, Jinan, 250010 China

**Keywords:** *Salmonella enterica* serovar Enteritidis, Shouguang chicken, Cecum, Transcriptome sequencing, miRNA

## Abstract

**Background:**

*Salmonella enterica*, serovar Enteritidis (SE) is a food-borne pathogen, which can cause great threat to human health through consumption of the contaminated poultry products. Chicken is the main host of SE. The mRNA and microRNA (miRNA) expression profiles were analyzed on cecum of Shouguang chicken via next-generation sequencing and bioinformatics approaches. The treated group was inoculated SE, and the control group was inoculated with phosphate buffer saline (PBS).

**Results:**

There were 1760 differentially expressed mRNAs in the SE-infected group, of which 1046 were up-regulated mRNA, and 714 were down-regulated mRNA. In addition, a total of 821 miRNAs were identified, and 174 miRNAs were differentially expressed, of which 100 were up-regulated and 74 were down-regulated. Functional enrichment of differentially expressed mRNAs was similar to miRNA target genes. The functional analysis results of differentially expressed mRNAs and miRNAs were performed. Immune-related processes and KEGG (Kyoto Encyclopedia of Genes and Genomes) pathways were enriched by up-regulated mRNA. The down-regulated mRNAs were enriched in tissue development and metabolic-related KEGG pathways. The functional analysis of up-regulated miRNA target genes was similar to the down-regulated mRNAs. The down-regulated miRNA target genes were enriched in metabolic-related GO (Gene Ontology) -BP (Biological process) terms and KEGG pathways. The overlap of the up-regulated mRNA and the up-regulated miRNA target genes (class I) was 325, and the overlap of the down-regulated miRNA target genes (class II) was 169. The class I enriched in the immune-related GO-BP terms and KEGG pathways. The class II mainly enriched in metabolic-related GO-BP terms and KEGG pathways. Then we detected the expression of mRNA and miRNA through qRT-PCR. The results shown that the expression of *HHIP*, *PGM1*, *HTR2B*, *ITGB5*, *RELN, SFRP1*, *TCF7L2*, *SCNN1A*, *NEK7,* miR-20b-5p, miR-1662, miR-15a, miR-16-1-3p was significantly different between two groups. Dual-luciferase reporter assay was used to detect the relationship between miR-20b-5p and *SCNN1A*. The result indicated that miR-20b-5p regulate immune or metabolic responses after SE infection in Shouguang chickens by directly targeting *SCNN1A*.

**Conclusions:**

The findings here contribute to the further analysis of the mechanism of mRNA and miRNA defense against SE infection, and provide a theoretical foundation for the molecular disease-resistant breeding of chickens.

## Background

*Salmonella* is an important food-borne zoonotic pathogen, which can directly or indirectly infect animals and further contaminates animal products such as meat, eggs, milk, etc., thereby posing a threat to human health [1]. Salmonellosis is an acute or chronic animal disease caused by certain specific serotypes of *Salmonella*, which has important public significance of health [2]. The bacterial serotype with the highest isolation rate in sick poultry is SE with an isolation rate of 37% [3, 4] which is a major cause of food-borne gastroenteritis. 40–80% of food poisoning incidents in developed countries such as the United Kingdom and the United States were caused by SE [5]. Chicken is the major reservoir of SE. It has been reported that the losses caused by egg-related salmonellosis have reached $44 million in Australia every year [6].

Transcriptomics, proteomics, metabolomics, etc., have become important research tools, among which transcriptomics has been applied earlier and has the most extensive [7]. Transcriptome sequencing is widely used in skeletal muscle of mice [8], spleen of chicken [9], fallopian tube of horse [10], etc.. The expression of *TLR1A* in the cecum tissue was significantly up-regulated on 7-days post infection with SE of White Leghorn Layer in our previous research [11]. The research that performed RNA-Seq from lung and spleen samples suggested that an early inactivation of important host genes could prevent an exaggerated immune response and/or viral replication, conferring resistance to HPAIV in chickens [12]. In addition, transcriptomic analysis revealed that more severe disease in line W chicken was associated with significant up-regulation of pathways involved in inflammation, cytoskeletal regulation by Rho GTPases, and Wnt signaling in the bursa, etc. [13] And another research focused on cecum of 1-day-old chickens infected with SE by RNA-seq, and 104 differentially expressed genes were identified, included *IL-22*, *IL-1β*, etc. inflammation-related genes [14]. The expression of *IL-17C*, *CIKS*, *TRAF6*, *NFkappaB*, *C/EBPbeta*, and inflammatory chemokines were significantly up-regulated in response to co-infection of *Mycoplasma gallisepticum* and *Escherichia coli* in chicken [15]. The study of CA09 virus infection in mice was mostly involved with genes related to the extracellular matrix (ECM), while the most significant differences after SD56 infection in mice were in immune-related genes [16].

miRNA is an endogenous single-stranded non-coding RNA molecule with a length of 18–26 nt, which is widely present in natural animals, plants and viruses. miRNA can bind to the 3’UTR region of the target gene and participates in the inhibition of mRNA degradation or translation [17], and then affect protein coding and expression [18, 19]. A large body of research results showed that autoimmune diseases and immune dysregulation are associated with different miRNA expression changes in the target cells and tissues of adaptive or innate immunity. Next-generation sequencing technology has been applied in the field of miRNA research. The splenocyte miRNA profile of pig infected with *T. gondii* revealed that the coordination of a large number of miRNAs regulates the host immune response during infection [20]. The studies on the expression profile of miR-155 suggest that the altered expression and function of miR-155 can mediate vulnerability to autoimmune diseases [21]. By inhibiting the expression of adipocyte-related miR-103, the expression of porcine precursor adipocyte differentiation-related genes were significantly reduced [22]. miR-125a is significantly different expressed in liver and adipose tissue, plays a regulatory role in the MAPK signaling pathway [23].

The genome-wide miRNA–mRNA interaction network following SE inoculation was not clear. In the current study, the interaction between miRNAs and mRNAs in cecum of Shouguang chicken following SE inoculation was analyzed using next-generation sequencing technology.

## Results

### Identification of differentially expressed mRNA and functional enrichment analysis

**Fig. 1:**
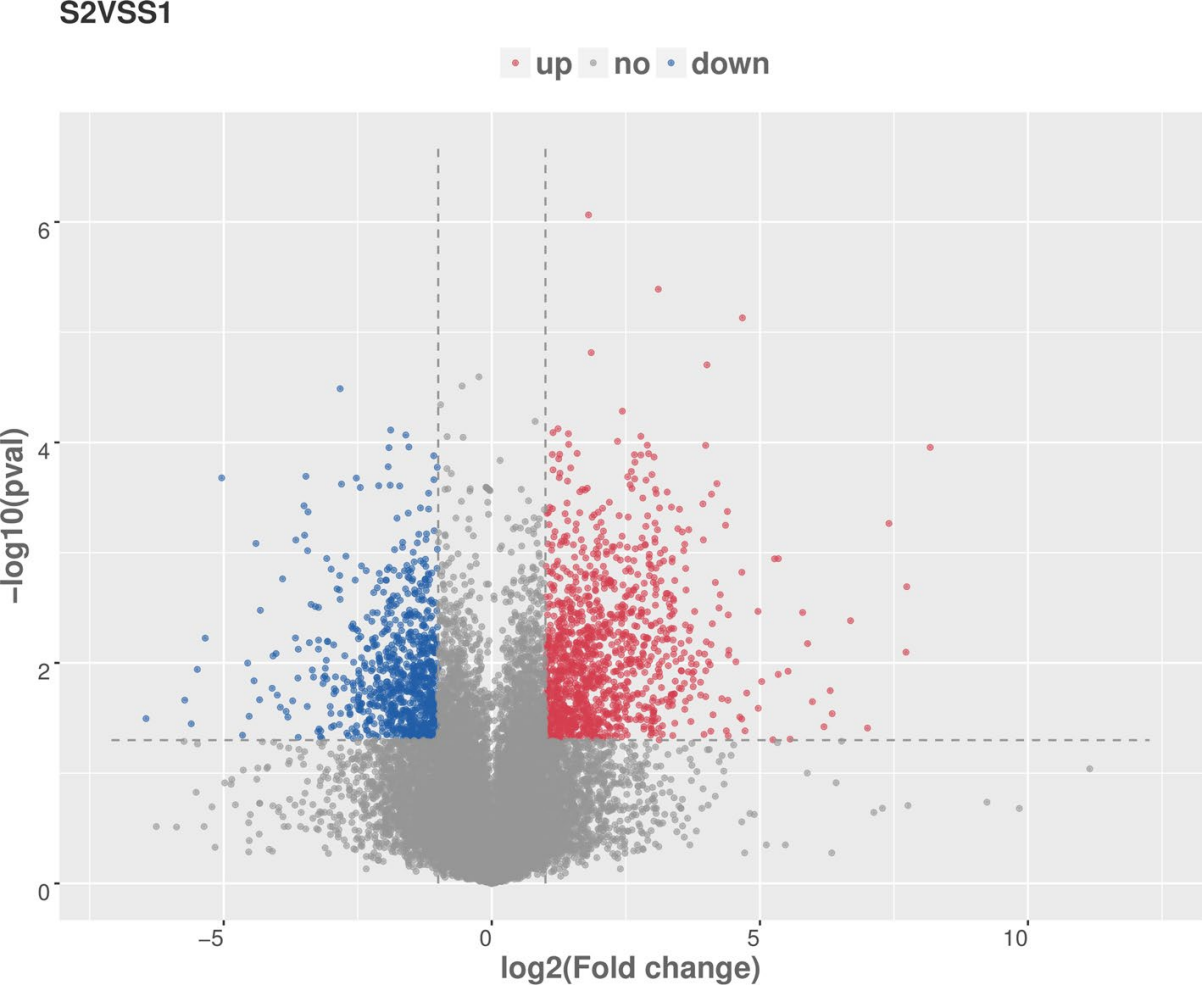
The volcano map of differentially expressed mRNA, gray colour represents non-differentially expressed mRNA, red represents up-regulated mRNA and blue represents down-regulated of differentially expressed mRNA

**Fig. 2:**
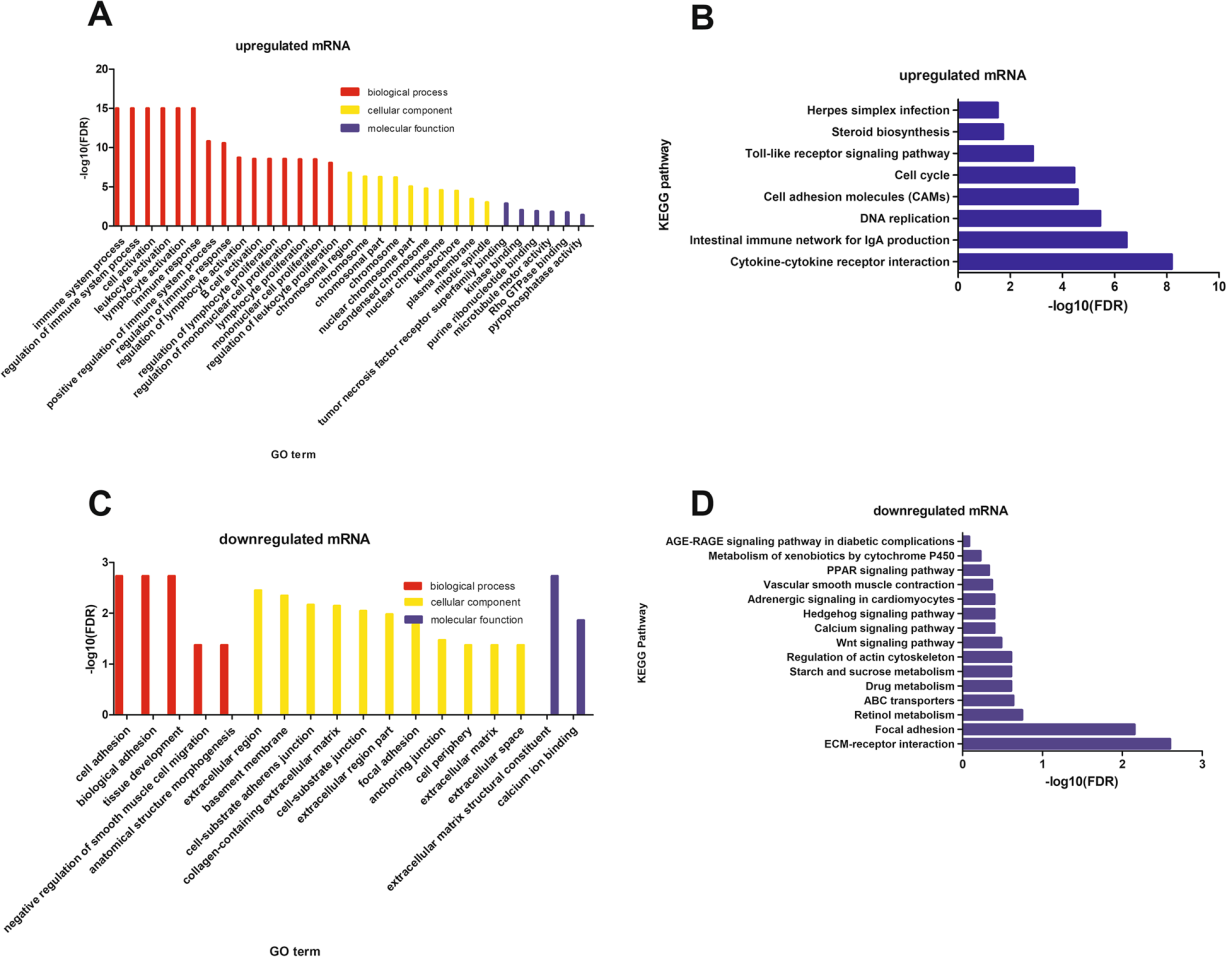
The results of functional enrichment analysis of differentially expressed mRNAs. **A** Enriched GO terms for up-regulated mRNAs. The x-axis was the GO terms and the y-axis was the –log10 FDR. **B** Enriched KEGG pathway for up-regulated mRNAs. The x-axis was –log10 FDR, and the y-axis was the KEGG pathway. **C** Enriched GO terms for down-regulated mRNAs. The x-axis was the GO terms and the y-axis was the –log10 FDR. **D** Enriched KEGG pathway for down-regulated mRNAs. The x-axis was –log10 FDR, and the y-axis was the KEGG pathway

In total, there were 1046 up-regulated mRNAs and 714 down-regulated mRNAs identified (|log2 (fold change) | ≥1, *P* < 0.05) (Fig. 1). The results of functional enrichment analysis of mRNAs were shown in Fig. 2. The mainly enriched GO terms (FDR < 0.0001) of up-regulated mRNAs was related to immune and inflammatory, such as immune system process, regulation of immune system process, immune response, positive regulation of immune system process and regulation of immune response, regulation of inflammatory response. Cell cycle related GO-BP terms were cell cycle DNA replication, positive regulation of cell cycle process. The defense response GO-BP term was defense response to bacterium (Fig. 2A). The enriched KEGG pathways included Cytokine-cytokine receptor interaction, Intestinal immune network for IgA production and Toll-like receptor signaling pathway (FDR < 0.05) (Fig. 2B).

The down-regulated mRNAs were intensively enriched in 5 GO-BP terms (FDR < 0.05)，involved cell adhesion, biological adhesion, tissue development, negative regulation of smooth muscle cell migration and anatomical structure morphogenesis (Fig. 2C). The top20 KEGG pathways were ECM-receptor interaction (FDR < 0.05), Drug metabolism, Wnt signaling pathway, Metabolism of xenobiotics by cytochrome P450, PPAR signaling pathway, Pentose phosphate pathway (FDR > 0.05) and other pathways (Fig. 2D)*.*

### Identification of differentially expressed miRNA, the prediction of miRNA target genes and functional enrichment analysis

**Fig. 3:**
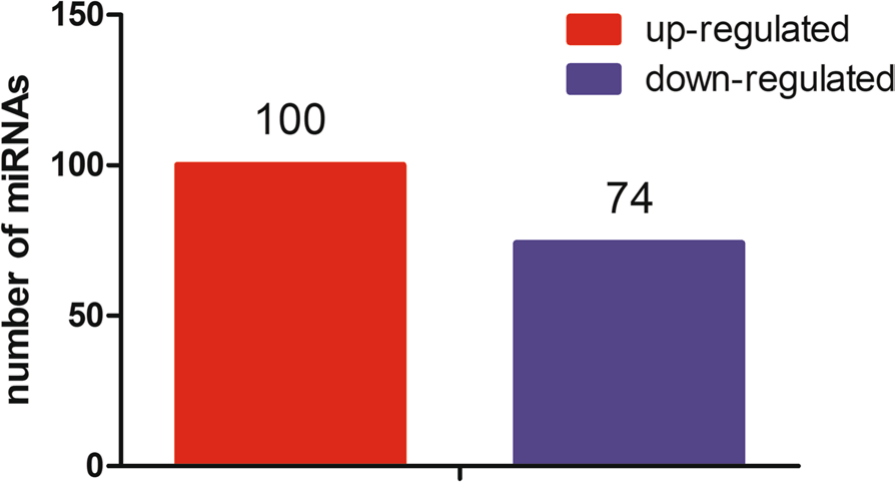
The results of differentially expressed miRNA. There were 100 up-regulated differentially expressed miRNAs, and 74 down-regulated differentially expressed miRNAs

**Fig. 4:**
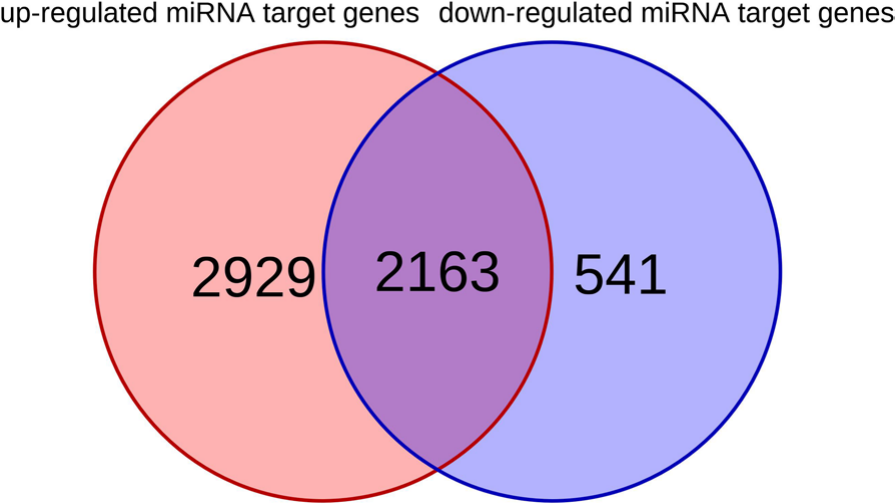
TargetScan and miRanda, were used to predict target genes of miRNAs, 2929 genes were targeted by up-regulated miRNAs alone, 541 genes were specifically targeted by down-regulated miRNAs, and 2163 genes were targeted by both

**Fig. 5:**
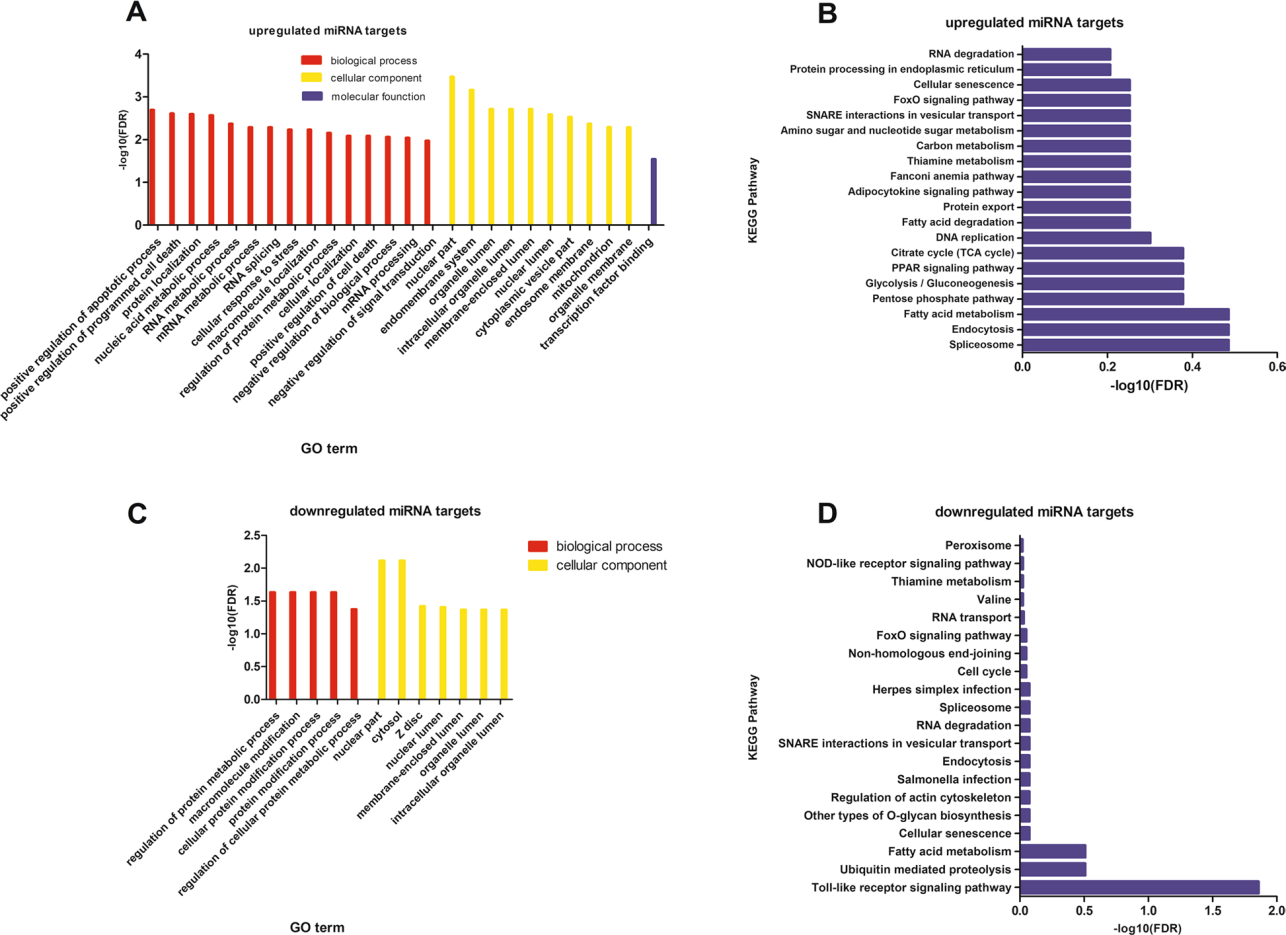
The results of functional enrichment analysis of differentially expressed miRNA target genes. **A** Enriched GO terms for up-regulated miRNA target genes. The x-axis was the GO terms and the y-axis was the –log10 FDR. **B** Enriched KEGG pathway for up-regulated miRNA target genes. The x-axis was –log10 FDR, and the y-axis was the KEGG pathway. **C** Enriched GO terms for down-regulated miRNA target genes. The x-axis was the GO terms and the y-axis was the –log10 FDR. **D** Enriched KEGG pathway for down-regulated miRNA target genes. The x-axis was –log10 FDR, and the y-axis was the KEGG pathway

There were 174 significantly expressed miRNAs, of those miRNAs, 100 miRNAs were up-regulated and 74 miRNAs were down-regulated (|log2 (fold change) | ≥1, *P* ≤ 0.05) (Fig. 3). The up-regulated miRNAs had 5092 target genes, the down-regulated miRNAs had 2704 target genes, and 2163 genes were targeted by both up-regulated miRNA and down-regulated miRNA (Fig. 4). We annotated functions for the up-regulated and down-regulated miRNAs target genes using WebGestalt. The up-regulated miRNA target genes were enriched a total of 90 GO terms (FDR < 0.05), including 62 biological process terms, 27 cellular component terms, and 1 molecular function. The enriched GO terms (Fig. 5A) mainly related to metabolism, apoptotic, transport, etc. The metabolism-related terms involved nucleic acid metabolic process, RNA metabolic process, mRNA metabolic process, regulation of protein metabolic process. However, KEGG pathway was not significant (FDR > 0.05), the top20 of KEGG pathway was shown in Fig. 5B, it was mainly related to endocytosis, fatty acid metabolism, pentose phosphate pathway, glycolysis/gluconeogenesis, Pentose phosphate pathway, PPAR signaling pathway, FoxO signaling pathway.

The down-regulated miRNA target genes were enriched in 5 GO-BP terms and 7 GO-CC (Cellular component) terms, and no GO-MF (Molecular function) terms was enriched (FDR < 0.05) (Fig. 5C). GO-BP terms involved the regulation of protein metabolism, macromolecule modification, cell protein modification process, protein modification process, and cell protein metabolism regulation, which were all related to protein synthesis and metabolism. The enriched KEGG pathways (Fig. 5D) of down-regulated miRNA target genes gathered in Toll-like receptor signaling pathway (FDR < 0.05), ubiquitin mediated proteolysis, *Salmonella* infection, NOD-like receptor signaling pathway and fatty acid metabolism (FDR > 0.05).

### The analysis combined differentially expressed mRNA and miRNA

**Fig. 6:**
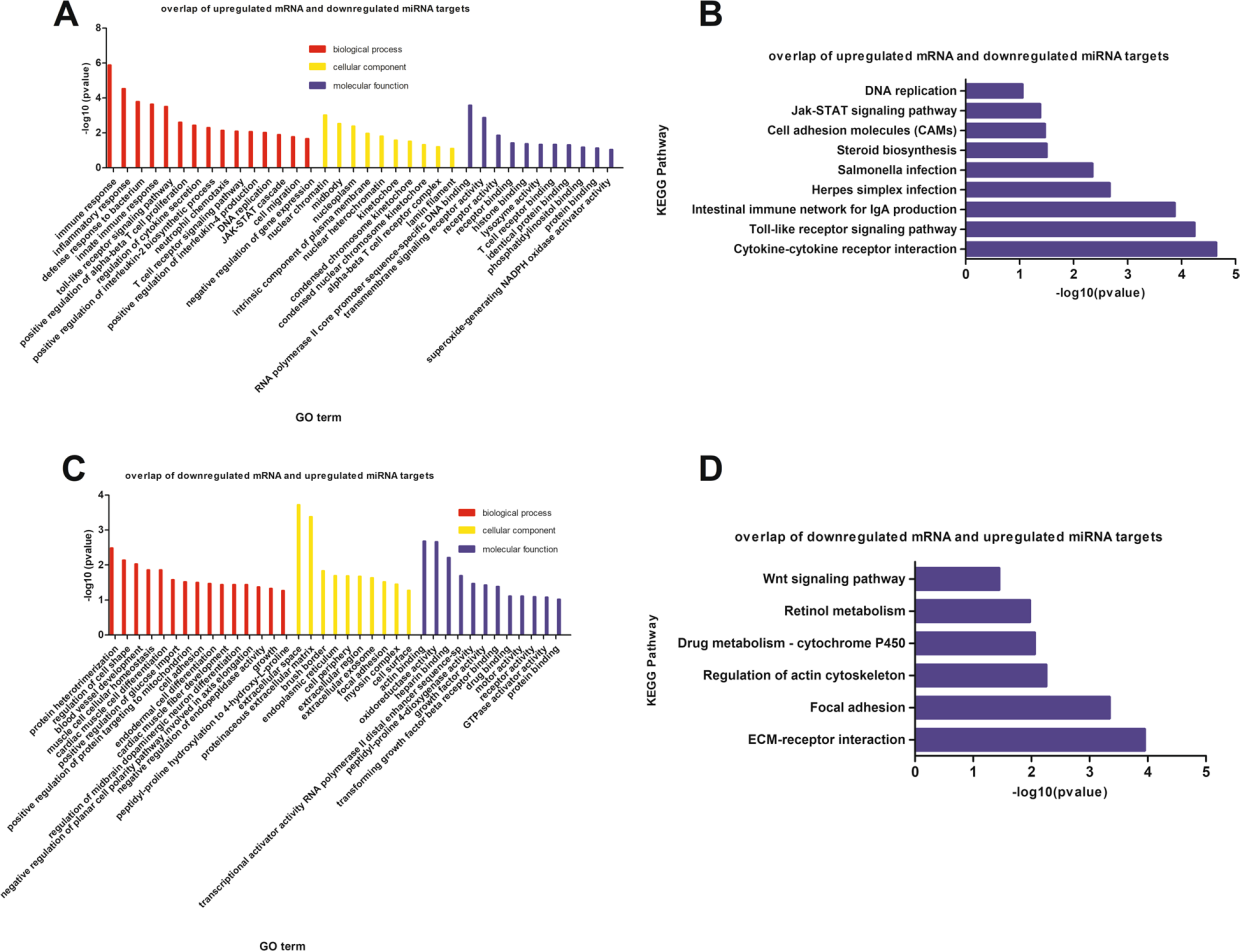
The results of functional enrichment analysis for the overlap of differentially expressed mRNA and miRNA target genes. **A** Enriched GO terms for the overlap of up-regulated mRNA and down-regulated miRNA target genes. The x-axis was the GO terms and the y-axis was the –log10 (*P* value). **B** Enriched KEGG pathway for the overlap of up-regulated mRNA and down-regulated miRNA target genes. The x-axis was –log10 (*P* value), and the y-axis was the KEGG pathway. **C** Enriched GO terms for the overlap of down-regulated mRNA and up-regulated miRNA target genes. The x-axis was the GO terms and the y-axis was the –log10 (*P* value). **D** Enriched KEGG pathway for the overlap of down-regulated mRNA and up-regulated miRNA target genes. The x-axis was –log10 (*P* value), and the y-axis was the KEGG pathway

We combined the differentially expressed mRNAs and miRNA target genes to explore the regulation in Shouguang chickens infected with SE. The overlap of the up-regulated mRNAs and the up-regulated miRNAs target genes was 325, and the overlap of the up-regulated mRNAs and down-regulated miRNAs target genes (class I) was 196. The overlap of the down-regulated mRNAs and up-regulated miRNA target genes (class II) was 221, and the overlap of the down-regulated mRNAs and down-regulated 1 was 111 (Table 1). For example, the down-regulated mRNAs, *SCNN1A* and *NEK7* were targeted by the up-regulated miRNAs, miR-20b-5p and miR-15a. And the up-regulated mRNA *CENPM* was targeted by the down-regulated miRNA miR-1662. Since mRNA and miRNA have potential negative regulatory relationship, we performed functional enrichment analysis of mRNA and miRNA with negative regulatory relationship (class I and class II). The enriched GO-BP terms of class I (*P* < 0.05) mainly included immune response, innate immune response, inflammatory response, leukocyte activation involved in inflammatory response, defense response to bacterium, toll-like receptor signaling pathway, regulation of cAMP metabolic process (Fig. 6A). There were 8 KEGG pathways (*P* < 0.05) have been enriched, including Cytokine-cytokine receptor interaction, Toll-like receptor signaling pathway, Intestinal immune network for IgA production, *Salmonella* infection, Steroid biosynthesis, Cell adhesion molecules and Jak-STAT signaling pathway (Fig. 6B), which were related to the internal regulation of chicken post-infection with SE.

**Table 1:** The results of correlated mRNA -miRNA

	No. of up-regulated mRNAs	No. of down-regulated mRNAs	No. of genes targeted by up-regulated miRNA	No. of genes targeted by down-regulated miRNA
No. of up-regulated mRNAs	1046	0	325	196
No. of down-regulated mRNAs		714	221	111
No. of genes targeted by up-regulated miRNA			5092	2163
No. of genes targeted by down-regulated miRNA			2163	2704

The enriched GO-BP terms (*P* < 0.05) of class II were mainly related to cell development and differentiation processes such as protein heterotrimerization, regulation of cell shape, cardiac muscle fiber development, and blood vessel development, etc. (Fig. 6C). A total of 6 KEGG pathways (*P* < 0.05) were significantly enriched, involved ECM-receptor interaction, Focal adhesion, Regulation of actin cytoskeleton, and Drug metabolism-cytochrome P450, Retinol metabolism, Wnt signaling pathway, which related to the metabolism (Fig. 6D).

### Interacting network of differentially expressed mRNA and differentially expressed miRNA target genes

**Fig. 7:**
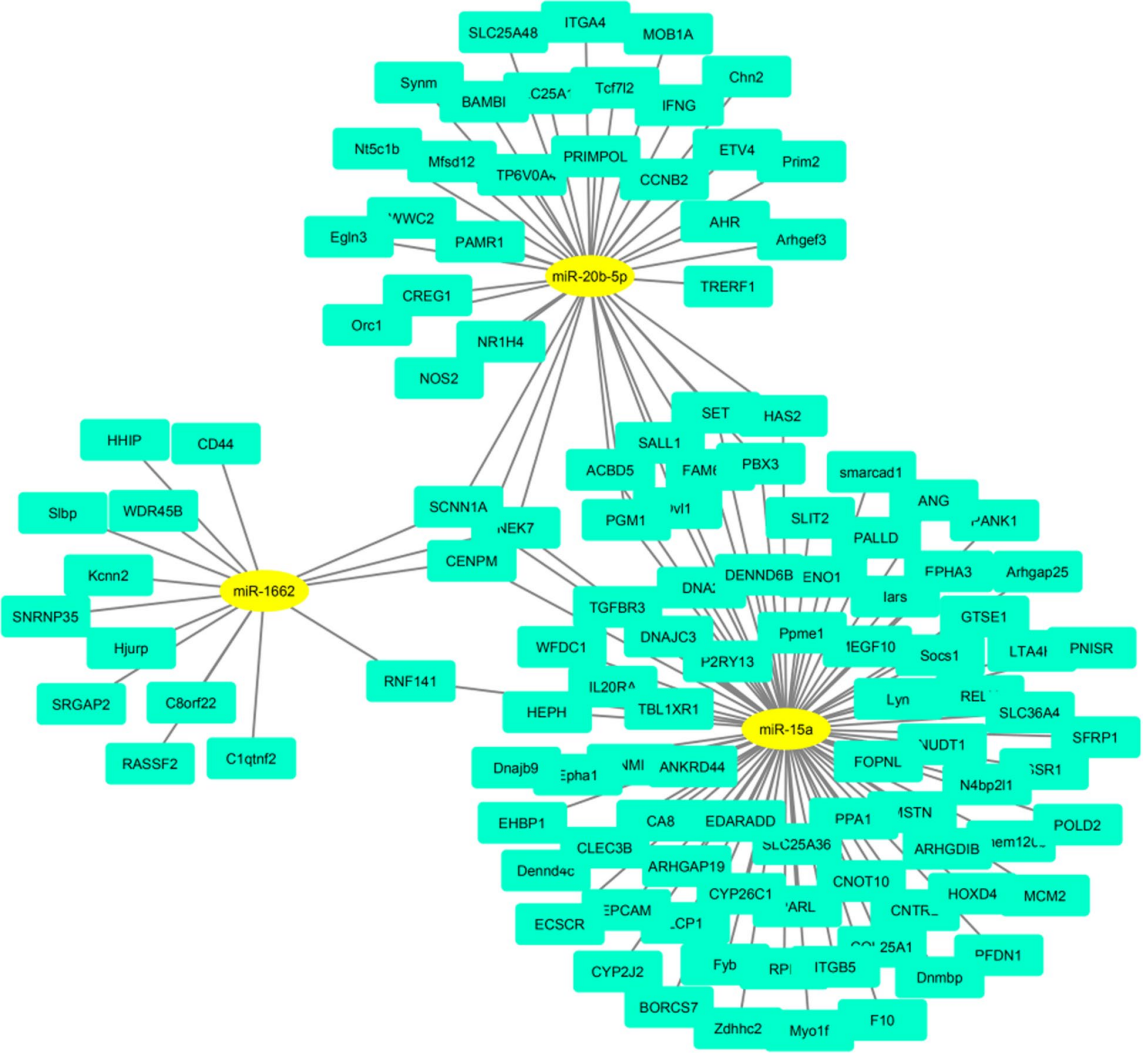
Interacting network of differentially expressed mRNA and differentially expressed miRNA target genes. The yellow bottom layer represented differentially expression miRNA, the blue bottom layer represented differentially expression mRNA, and the line indicated the relationship between the two mRNA and miRNA. *CENPM*, *SCNN1A*, and *NEK7* were simultaneously targeted by miR-20b-5p, miR-15a and miR-1662

We selected some differentially expressed mRNAs and miRNAs (*P* < 0.05) randomly to draw the interaction network with Cytoscape, version 3.8 (Fig. 7). There were 12 target genes were both targeted by miR-15a and miR-20b-5p, included *PGM1*, *CENPM*, *SCNN1A*, and *NEK7*. There were 4 target genes *RNF141*, *CENPM*, *SCNN1A* and *NEK7* both targeted by miR-15a and miR-1662. *CENPM*, *SCNN1A*, and *NEK7* were targeted by miR-1662, miR-15a, miR-20b-5p.

### Verification of differentially expressed mRNA and miRNAs

**Fig. 8:**
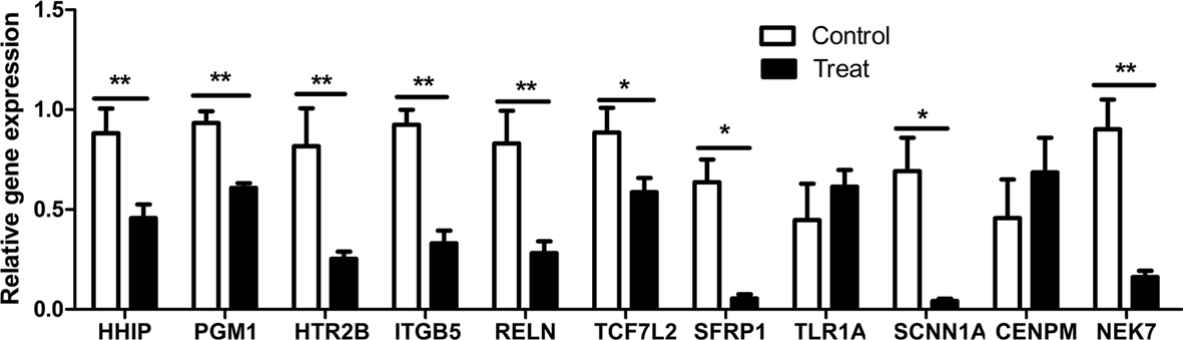
Relative expression of 11 differentially mRNAs. *β-actin* was the internal reference. Note: ** represent an extremely significant difference (*P* < 0.01); * represent a significant difference (*P* < 0.05), compared with the control group

**Fig. 9:**
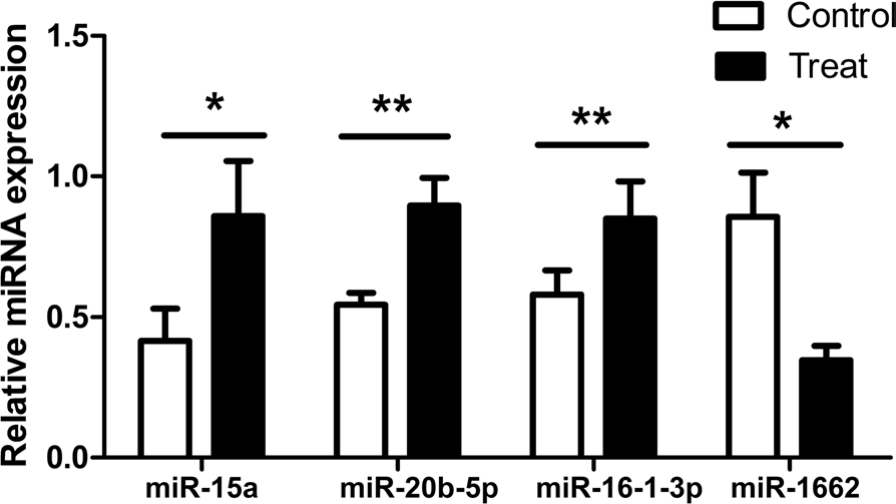
Relative expression of 4 differentially miRNAs. *U6* was the internal reference. Note: ** represent an extremely significant difference (*P* < 0.01); * represent a significant difference (*P* < 0.05)

The relative expression of 11 mRNAs and 4 miRNAs were validated through quantitative Real-Time PCR (qRT-PCR). The results (Fig. 8) showed that 9 mRNAs (*HHIP*, *PGM1*, *HTR2B*, *ITGB5*, and *RELN* (*P* < 0.01), *TCF7L2*, *SFRP1*, *SCNN1A* and *NEK7* (*P* < 0.05)) were differentially down-regulated, *CENPM* and *TLR1A* were up-regulated, which consistent with the trend of RNA-seq (*P* > 0.05). miR-20b-5p, miR-16-1-3p (*P* < 0.01) and miR-15a (*P* < 0.05) were significantly up-regulated, and miR-1662 (*P* < 0.05) was down-regulated in the treated group (Fig. 9). The result was consistent with the miRNA-seq. According to the results, we can see that the expression trend of miR-1662 is opposite to that of *CENPM* and *TLR1A*, and the expression trend of miR-20b-5p, miR-16-1-3p and miR-15a is opposite to that of the other 9 differentially expressed mRNAs.

### Validation of targeting relationships between *SCNN1A* and miR-20b-5p using dual-luciferase reporter assay

**Fig. 10:**
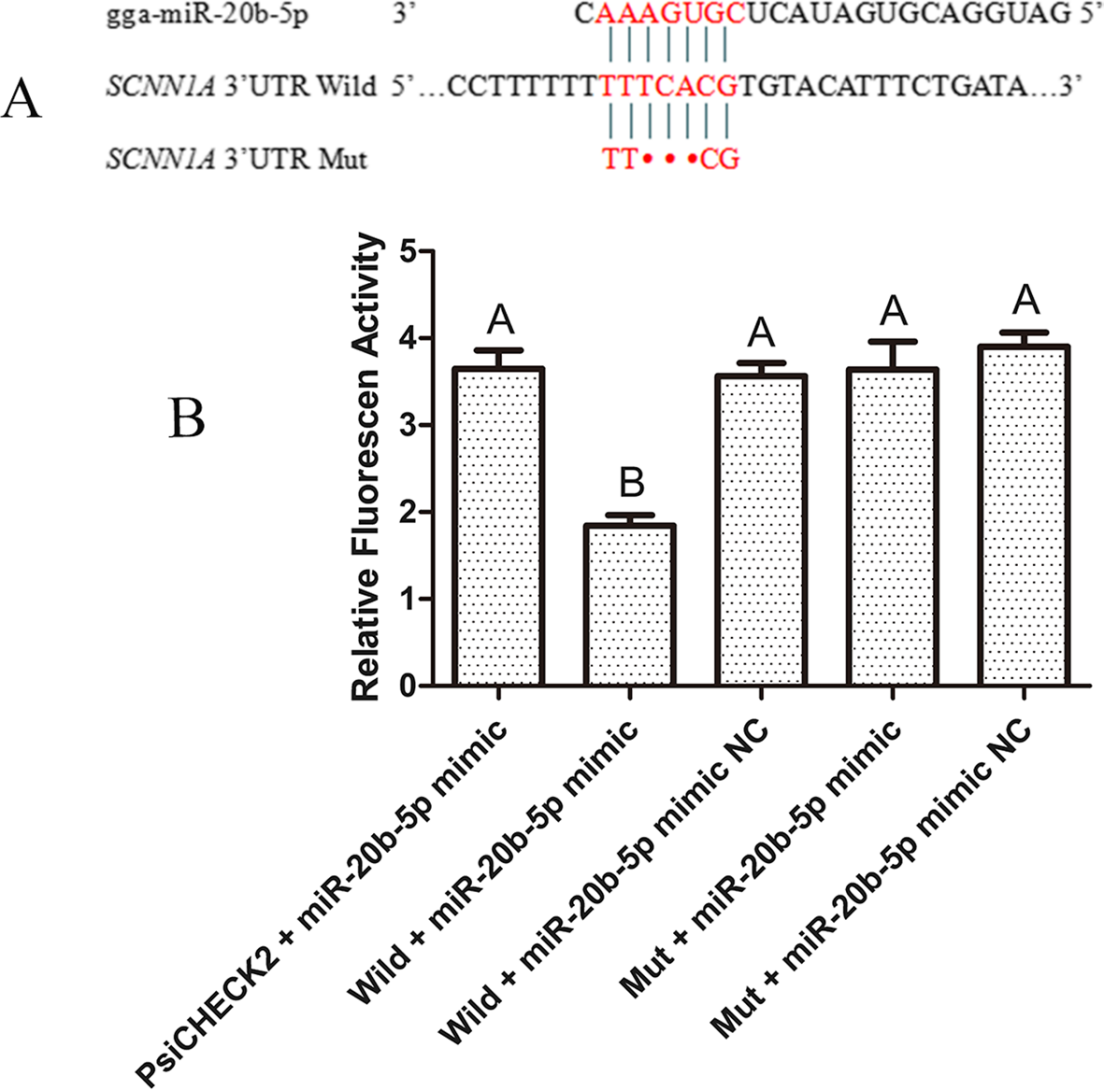
The results of dual-luciferase reporter system. **A** The sequences of wild type and mutant dual-luciferase reporter vector. Red nucleotides represented bind sites between miR-20b-5p and *SCNN1A* 3’UTR. **B** The relative luciferase activity of 5 groups. The relative luciferase activity of miR-20b-5p mimic + wild group was significantly lower than the other groups

The binding sites of miR-20b-5p and wild-type/mutant target sequences of *SCNN1A* into psiCHECK2 was showed in Fig. 10A. The dual-luciferase activity among 4 groups (psiCHECK2 + miR-20b-5p mimics, miR-20b-5p mimics NC + Wild, miR-20b-5p mimics/NC + Mut) was no significant difference, but the miR-20b-5p mimic + wild group was significantly lower than the other groups. The result indicated that miR-20b-5p can bind to the sequence of the 3’UTR of *SCNN1A* on the wild-type vector, thus reducing the luciferase acsetivity (Fig. 10B).

## Discussion

### Regulation of mRNA in the response to SE infection in Shouguang chicken

Transcriptome sequencing can accurately and efficiently obtain almost all the transcripts of a specific tissue at a certain period of time, and deeply excavated the subtle changes in the differential expression of each gene in the tissue or cell [24]. In current study, the KEGG pathways of up-regulated mRNAs had been enriched were mainly immune-related processes such as immune response, the intestinal immune network that produces immunoglobulin A, and Toll-like receptor signaling pathway. Researchers have pointed out that SE infection could active the immune response of body. Matulova’s research results showed that the expression of immunoglobulin A, G and other related genes in White Leghorn Layer chickens and other chicken breeds also increased significantly after infection, which is consistent with this experiment [14]. Up-regulated mRNAs were significantly enriched in the Toll-like receptor signaling pathway in our study. Toll-like receptors (TLRs) were a type of pattern recognition receptors that could specifically recognize pathogen-related molecular patterns (PAMPs) [25]. As an important component of the body’s immune system, the body’s immune process was regulated by TLRs while activated innate immunity [26]. Different types of TLRs were expressed in the spleen, liver, lungs, intestines and other different tissues of the body, and were mainly distributed in various immune cells such as B cells, T cells and dendritic cells in various tissues [27–32]. Compared to the control group, the expression of *TLR1A*, *TLR1B*, *TLR2A* and *TLR2B* in the treated group were significantly up-regulated through RNA-seq in our research, which was consistent with Mitra’s research of *TLR1B*, *TLR2B* and *TLR4* showed a continuous up-regulation in the cecum of chickens during infection or vaccination, followed by challenge with virulent parasites [33]. In addition, the expression of *TLR1A* in the cecum tissue was significantly up-regulated on 7-days post infection with SE of White Leghorn Layer in our previous research [11]. Therefore, we suspected that the Toll-like receptor signaling pathway played a potential role in chicken infection with SE. We found that the GO terms and KEGG pathways enriched by down-regulated genes were closely related to the development and metabolism of the body, such as tissue development, Wnt signaling pathway, and cytochrome P450 metabolism. The Wnt signaling pathway originated from int-like genes in Drosophila, which could influence cell differentiation and proliferation through signal transduction, thereby regulating growth and development [34]. The TCF7L2 protein was a key transcriptional effector of the Wnt signaling pathway, which was an important developmental pathway that negatively regulates adipogenesis [35, 36]. Inactivation of TCF7L2 protein by removing the high-mobility group (HMG)-box DNA binding domain in mature adipocytes in vivo led to whole-body glucose intolerance and hepatic insulin resistance. This phenotype was associated with increased subcutaneous adipose tissue mass, adipocyte hypertrophy, and inflammation [35]. In this work, the *TCF7L2* gene was significantly down-regulated, which was consistent with Chen’s research. We speculated that the development of adipose tissue in Shouguang chickens infected with SE was negatively affected, which in turn caused the growth and development of the body to be hindered and an inflammatory response occurred. At the same time, the appearance of glucose intolerance would also affect the glucose metabolism process. Phosphoglucomutase (PGM) catalyzed the interconversion of glucose-1 phosphate (Glc-1P) and glucose-6 phosphate (Glc-6P) and therefore, it played a fundamental role in glycolysis, glycogenesis, and glycogenolysis [37]. In the present experiment, the expression levels of *PGM1*, *PGM3*, and *PGM5* were all significantly down-regulated. It can be seen that the synthesis of phosphoglucomutase in Shouguang chickens infected with SE was inhibited. *PGM1* deficiency resulted in a mixed phenotype of a Glycogen Storage Disorder and a Congenital Disorder of Glycosylation (CDG) [38]. The symptoms of CDG included muscle cramps, rigid limbs after exercise, slow energy recovery, etc. This might be one of the reasons for the malaise and loss of appetite in chickens infected with SE. Therefore, we boldly speculated that at the mRNA level, the infection of SE mainly caused immune responses in Shouguang chickens. At the same time, it also affected the growth, development and metabolism of the body by regulating the differentiation of adipocytes and the process of glucose metabolism.

### Regulation of mRNA in the response to SE infection in Shouguang chicken

Next Generation Sequencing can be performed on any tissue/organ without prior information about sequences or secondary structure. It can also detect miRNAs with very low copy numbers, which reflect either a low expression level in the tissue or a contribution from minor cell types such as blood or adipose or connective tissues in the biopsy specimen [39–41]. In this experiment, the number of up-regulated miRNAs was more than down-regulated miRNAs, which was consisted with the other researches of miRNA sequencing in chicken [42, 43]. The pathways for up-regulated miRNA target genes were cell apoptosis, nucleic acid metabolism, regulation of protein metabolism, glycolysis/gluconeogenesis, PPAR signaling pathway and other metabolic-related processes, the down-regulated miRNA target genes were mainly enriched in Toll -like receptor signaling pathway, which was the immune-related process. In previous researches, miRNAs could interact with key genes in metabolism-related pathways, for example, PPAR receptors played a critical role in metabolic disorders, affected glucose and lipid metabolism [44, 45]. The PPAR signaling pathway was enriched by up-regulated miRNA targets in our work, and some researches showed that the PPAR pathway was widely involved in lipid metabolism, immune process and inflammatory response process [46–49]. Zhao analyzed the expression of more than 200 miRNAs in islets, liver, and adipose tissues of mice, and found that more than half of miRNAs were expressed in the above-mentioned tissues, and there was a significant difference between diabetic mice and normal mice [50], which also confirmed that miRNAs were played an important role in the regulation of the body’s metabolic process. miR-15a was one of the miRNAs who related to metabolism. The overexpression of miR-15a would inhibit the proliferation of pre-adipocytes and increased their size, thereby regulated the lipid metabolism process [51]. The expression level of miR-15a were changed accompanied by insulin synthesis, indicating that miR-15a could affect the glucose metabolism process by regulating the insulin synthesis process [52]. Similarly, miR-20b-5p also played an important role in the metabolic process of chicken. In recent studies, miR-20b-5p overexpression increased basal glycogen synthesis in human skeletal muscle cells, and insulin-stimulated glycogen accumulation by reducing the abundance of AKTIP [53]. In addition, there were reports found that miR-20b-5p promoted myoblast differentiation and repressed myoblast proliferation by directly binding the 3′ UTR of E2F transcription factor 1 (E2F1) mRNA [54]. Therefore, miR-20b-5p could regulate the growth and development to a certain extent. In this experiment, the expression levels of miR-15a and miR-20b-5p in the treatment group were significantly up-regulated compared to the control group. Therefore, we inferred that the lipid metabolism and glucose metabolism were affected by the infection of SE in Shouguang chickens, and it would also have an impact on growth and development. The results of functional enrichment also involved the Toll-like receptor signaling pathway, which was closely related to miR-1662. We speculate that miR-1662 was linked to the Toll-like receptor signaling pathway through its target gene *TLR1A*. Our previous studies have shown that miR-1662 responds to SE infection in chickens by targeting *TLR1A* [11]. This phenomenon was consistent with the results of this study. We believed that the immune response was activated after being infected with SE in Shouguang chickens, and miR-1662 played an important role in defending against SE infection. Therefore, we had evidence to prove that the infection of SE could cause the change expression level for miRNAs, which related to immune, metabolic, growth and development, and then regulated the biological process of the body in further.

### The interacted regulation of mRNA and miRNA in the response to SE infection in Shouguang chicken

miRNAs are small non-coding RNA molecules, which are key players in gene expression regulation [55]. 3′-UTRs of mRNA are primarily targeted by miRNA molecules that form complex gene regulatory networks [56]. Generally, the expression trend of miRNA and its target gene was opposite. In this study, the two groups of mRNA and miRNA with opposite expression trends for function enrichment analysis, that were class I and class II. Toll-like receptor signaling pathway and the JAK-STAT signaling pathway were enriched in class I, which related to the body’s innate immunity. JAK signaling also regulated development and maturation of cells of the innate and adaptive immune systems [57, 58]. The binding of Toll-like receptors with specific adaptor proteins in the Toll-like receptor signaling pathway could promote inflammatory response and active immune response [25, 59, 60]. Herein, the expression of *TLR1A* was significantly up-regulated, and the expression of miR-1662 was significantly down-regulated. Preliminary studies had shown that *TLR1A* was targeted by miR-1662, and *TLR1A* was an important gene in the Toll-like receptor signaling pathway. We believed that both the Toll-like receptor signaling pathway and the JAK-STAT signaling pathway play an important role in the regulation of the immune response in Shouguang chickens infected with SE, and the Toll-like receptor signaling pathway might be regulated by down-regulated miR-1662 and up-regulated *TLR1A* together, which contributed to defend against the infection of SE. The results of functional enrichment analysis of class II group involved metabolic-related and development and growth process, e.g., Wnt signaling pathway, which related to the differentiation of the muscle and adipose tissue cells, and it also affected the development of pancreatic islets [34]. The up-regulated miRNA, miR-15a and miR-20b-5p, both played a role in the development and metabolism of the body. Mainly The glucose metabolism process and the development and differentiation of precursor adipocytes were regulated of miR-15a by inhibiting the formation of insulin [52], and miR-20b-5p promoted hepatocellular carcinoma cell proliferation, migration and invasion by down-regulating *CPEB3* [61]. In our research, miR-20b-5p regulate immune or metabolic responses after SE infection in Shouguang chickens by directly targeting *SCNN1A* The down-regulated genes *PGM1* and *TCF7L2* was the target gene of miR-15a and miR-20b-5p, respectively, PGM1 (Phosphoglucomutase 1) encoded the metabolic enzyme that interconverts glucose-6-P and glucose-1-P, and it could modulated lipolysis and glycometabolism [62, 63]. *TCF7L2* was a key mediator of the evolutionary conserved canonical Wnt signaling pathway, additionally, TCF7L2-mediated and calcineurin/nuclear factor of activated T cells-mediated target genes were involved in insulin synthesis and secretion, insulin degradation, pancreatic beta-cell growth and regeneration, and functional application of pancreatic beta-cells [64–66]. Based on the above results, we found that miR-20b-5p was up-regulated and *TCF7L2* expression was down-regulated in Shouguang chickens in the treatment group, both of which affected the Wnt signaling pathway and the growth and development process. Similarly, the up-regulation of miR-15a and the down-regulation of the expression of *PGM1* hinder the transfer of glucose-6-P and glucose-1-P in the process of glucose metabolism, leading to abnormal metabolic processes in the birds.

## Conclusion

In conclusion, the infection of SE shown the changes in the expression of mRNA and miRNA in the cecum tissue of Shouguang chickens. Toll-like receptor signaling pathway, Wnt signaling pathway, PPAR signaling pathway, Fatty acid metabolism, glucose metabolism related pathways all played a crucial role in SE infection. *PGM1*, *TCF7L2*, *SCNN1A*, *NEK7*, miR-15a, miR-20b-5p and miR-1662 were differentially expressed after SE infection. The findings here contribute to the further analysis of the mechanism of mRNA and miRNA defense against SE infection, and provides a theoretical foundation for the molecular disease-resistant breeding of chickens.

## Methods

### The current study was carried out in compliance with the ARRIVE guidelines

All animal procedures were approved by the Shandong Agricultural University Animal Care and Use Committee (Approval Number: # SDAUA-2018-058) and performed in accordance with China animal welfare laws.

### Animal inoculation and sample collection

In the current study, Shouguang chicken, a Chinese native breed was used. A total of 70 SE-negative 2 day-old chickens were randomly divided into two groups (40 chicken in treated group, 30 chicken in control group). Chickens in treated and control groups were raised in two separate incubators with the same environmental conditions and free access to sterilized feed and water. The SE (CVCC3377) used for inoculation was provided by the China Veterinary Culture Collection Center (http://cvcc.ivdc.org.cn/). Each chicken in the treated group was orally inoculated with 0.3 mL of 3.54 × 10^8^ colony-forming units (cfu)/mL SE inoculant. Each chicken in the control group was inoculated with 0.3 mL sterile PBS. On day 3 after inoculation, six chicken in each of treated group and control group were euthanized by cervical dislocation after anesthetized with carbon dioxide (CO_2_), cecum was collected from each bird, snap frozen and kept in − 80 °C.

### Library construction and sequencing

Total RNA from each cecum was extracted using Trizol reagent (Invitrogen, CA, USA) following the manufacturer’s procedure. The quantity, purity and integrity of total RNA were analyzed using NanoDrop 2000 (Denovix, USA) and Bioanalyzer 2100 (Agilent, CA, USA) with RIN number > 7.0. mRNA was obtained using poly-T oligo from 1 μg total RNA in each sample. Then the mRNA was fragmented using NEBNext® Magnesium RNA Fragmentation Module (NEB, USA) under 94 °C 5-7 min. Then the cleaved mRNA fragments were reverse-transcribed to create the cDNA library in accordance with the protocol for the RNA-Seq sample preparation kit (Illumina, San Diego, USA). The libraries were sequenced using Illumina Hiseq 2500 platform following the manufacturer’s protocol at the Genergy Bio-Technology Co., Ltd. (Shanghai, China).

Approximately 1 μg of total RNA per sample was used for small RNA library construction according to the protocol of the Illumina small RNA Sample preparation kit. The 5′ adaptors and 3′ adaptors were ligated using T4 RNA ligase. And the 3′ and 5′ adapters-ligated RNA was performed to RT-PCR for amplification. The cDNA fragments located at 145–160 bp were extracted with polyacrylamide gel electrophoresis and purified via PAGE gel. The enriched cDNA library passed quality control with 2100 Bioanalyzer High Sensitivity DNA chip and KAPA Quantitative kit (Cat no. KK4602) was sequenced using the Hiseq 2500 sequencing platform (Illumina, San Diego, CA, USA) by Genergy Bio-Technology Co., Ltd. (Shanghai, China).

### RNA-seq data analysis

After removed the low quality bases and undetermined bases, we used HISAT2 software (https://daehwankimlab.github.io/hisat2/) to map reads to the UCSC (http://genome.ucsc.edu/) chicken reference genome. The mapped reads of each sample were assembled using StringTie (http://ccb.jhu.edu/software/stringtie/). StringTie and Ballgown (http://www.bioconductor.org/packages/release/bioc/html/ballgown.html) were used to estimate the expression levels of all transcripts and perform expression level for mRNAs by calculating Fragments Per Kilobase of transcript per Million fragments mapped (FPKM). The differentially expressed mRNAs were selected with the criteria of |log2 (fold change) | ≥ 1 and *P* < 0.05 using R package edgeR (https://bioconductor.org/packages/release/bioc/html/edgeR.html).

### miRNA-seq data primary analysis and target genes prediction

Raw reads were subjected to Bowtie tool to remove adapter dimers, junk, low complexity, common RNA families (rRNA, tRNA, snRNA, snoRNA) and repeats. The remaining reads were used to detect known miRNA by comparing with known miRNAs from miRBase. Differentially expression of miRNAs based on normalized counts was analyzed using Student t test. The significance threshold was |log2 (fold change) | ≥ 1 and *P* < 0.05. Target Scan 7.0 and MiRanda 3.3a were used to predict the target genes of miRNAs, and to identify miRNA binding sites. The overlapped genes predicted by both software were defined as the target genes of miRNA.

### Functional enrichment analysis

Functional annotation for mRNA and the predicted miRNA target genes were performed through the WebGestalt [67]. The GO terms and KEGG Pathway [68–70] enrichment analyses of the overlapped mRNA and miRNA target genes were performed using DAVID [71], version 6.7. All figures were drew using GraphPad Prism version 5.0 [72].

### Verification of differentially expressed mRNAs and miRNAs by quantitative real-time PCR

Every total RNA sample was reverse transcribed into cDNA with PrimeScript™ RT reagent Kit with gDNA Eraser (Perfect Real Time) (Takara, Dalian, China) for mRNA and SYBR™ PrimeScript miRNA RT-PCR Kit (Takara, Dalian, China) for miRNA, respectively. The primers (Table 2) were designed using DNAMAN and synthesized by Tsingke Biotechnology Co., Ltd. (Qingdao, China). SYBR® Green Premix Pro Taq HS qPCR Kit and SYBR® Green Premix Pro Taq HS qPCR Kit II (AG, Changsha, China) were used to detect the expression level of mRNA and miRNA. *β-actin* and *U6* were used as the housekeeping genes. The qRT-PCR performed using the Roche LightCycler®96 (Roche Diagnostics, Shanghai, China). The data were analyzed using the general linear model procedure of SAS 9.2 software (SAS Institute, Cary, NC). The relative expression was calculated using the 2^−ΔΔCt^ method. *P* < 0.05 was considered as significant.

**Table 2:** Primer sequences and the length of PCR products

Name	Accession No.	Primer Sequences (5’-3’)	Product length/bp
*β-actin*	NM_205518	F: TGCTGTGTTCCCATCTATCG	150
		R: TTGGTGACAATACCGTGTTCA	
*TLR1A*	NM_001007488	F: ATGACCAGCCGTATGAAATC	261
		R: TGCGTTCCGCTCAAGTC	
*PGM1*	NM_001038693	F: ACCACCTCAAGATTCGCA	130
		R: CCACCGAAGTCCTCCAG	
*TCF7L2*	NM_001206510	F: TGCGAAGAGGCAAGATG	102
		R: GATCCGTTGGGCAGATAC	
*RELN*	NM_001305123	F: TCGCAGACCTTCCCAAT	112
		R: GAACCCCACAGCCAAAG	
*HHIP*	XM_015276719	F: GATGGTGGTGTATGCTTTCC	114
		R: GTGCTTTCTGCTGATCTCTTC	
*HTR2B*	NM_001290547	F: CGGTAGCAGAACCCAAAG	123
		R: CCAGGATGACCAGGATGT	
*ITGB5*	NM_204483	F: CAGAGGGCGGTTTCGAT	132
		R: CCAGCTTCCCATCCAGAG	
*SFRP1*	NM_204553.5	F: CTGTGTGCCAGTGAGTTTGC	125
		R: AGGTTCTTCTTGCGGATGGG	
*SCNN1A*	NM_205145	F: AAGGAAGATGAGAGGGAGGG	266
		R: GCAGGGAAAGTCAGCCTATC	
*CENPM*	NM_001044638	F: CAACATCAACATCCACCTTG	230
		R: TGGCATTCATCTCTACGCT	
*NEK7*	NM_001031264	F: GCTGCCTGCTGTATGAGAT	192
		R: AGGTTATGTCTGGTCGCTTC	
gga-miR-15a	MIMAT0001117	TAGCAGCACATAATGGTTTGT	
gga-miR-20b-5p	MIMAT0001411	CAAAGTGCTCATAGTGCAGGTAG	
gga-miR-16-1-3p	MIMAT0026500	CCAGTATTAACTGTGCTGCTGAA	
gga-miR-1662	MIMAT0007543	TTGACATCATCATACTTGGGAT	
U6		CCAAGGATGACACGCAAA	

### Dual-luciferase reporter assay

*SCNN1A* and miR-20b-5p were selected for targeting relationship in chicken fibroblasts DF1 by a dual-luciferase reporter assay. The wild type vector psiCHECK2-SCNN1A-3’UTR-Wild and the mutant vector psiCHECK2-SCNN1A-3’UTR-Mut, were synthesized by Tsingke Biotechnology Co., Ltd. (Qingdao, China), and miR-20b-5p mimic/negative control (NC) was synthesized by Jiangsu Saisuofei Biotechnology Co., Ltd. (Wuxi, China). In 24-well plates, DF1 cells were cultured to approximately 70% confluence and then co-transfected with either wild type or mutant luciferase reporter vector and either mimic miRNAs or NC with Lipofectamine® LTX and Plus™ Reagent (Invitrogen, Carlsbad, CA, USA). After 48 h, luciferase activity was measured using Dual-Luciferase® Reporter Assay System Kit (Promega, USA) and normalized to the activity of renilla luciferase.

## Data Availability

The raw sequence data of RNA-seq and miRNA-seq have been submitted to the NCGC Genome Sequence Archive with accession number CRA006250 (https://ngdc.cncb.ac.cn/gsa/s/l5v7JHV2).

## Abbreviations

SE: *Salmonella enterica* serovar Enteritidis
miRNA: microRNA
PBS: Phosphate buffer saline
FPKM: Fragments Per Kilobase of transcript per Million fragments mapped
GO: Gene Ontology
BP: Biological process
MF: Molecular function
CC: Cellular component
KEGG: Kyoto Encyclopedia of Genes and Genomes
NC: Negative control
